# Efficacy and Safety of Artiss Fibrin Tissue Sealant Use in Rhytidectomy: A Review of 120 Cases

**DOI:** 10.1055/s-0037-1599237

**Published:** 2017-04-07

**Authors:** Kyle T. Yamamoto, Louis M. DeJoseph

**Affiliations:** 1 Premier Image Cosmetic and Laser Surgery, Atlanta, Georgia; 2 Sierra Nevada Cosmetic and Laser Surgery, Reno, Nevada

**Keywords:** facelift, Artiss, hematoma, fibrin, Tisseel

## Abstract

Hematoma formation has persisted as the most common complication in rhytidectomy. The objective of this study is to determine the efficacy and safety of Artiss (Baxter) for use in rhytidectomies. In addition, we determine the use of fibrin tissue sealants by facial plastic surgeons. In this retrospective chart review, 120 patients in a single private practice were identified who underwent a rhytidectomy from August 2013 to January 2015 by a single facial plastic surgeon. The last 60 rhytidectomies performed with Tisseel (Baxter) were compared with the first 60 rhytidectomies performed with Artiss.

All perioperative or postoperative complications were identified and recorded, focusing on the incidence of hematoma. In addition, a six-question survey was created and sent to all members of the American Academy of Facial Plastic and Reconstructive Surgery. Results of the survey were recorded and analyzed for trends or patterns in the data. In total, 120 patients were assessed. In the Tisseel group, two complications of fluid collection requiring needle aspiration were recorded. No other complications were found. In the Artiss group, 10 complications were recorded, including 9 fluid collections requiring needle aspiration and 1 hematoma. In total, 179 members of the American Academy of Facial Plastic and Reconstructive Surgery completed the six-question survey. Of all respondents, 61 (34%) use tissue sealants for rhytidectomies, whereas 118 (66%) do not. Artiss is efficacious and safe for use in rhytidectomies. Its use obviates the need for surgical drains, and complications are minimal and similar in rate to the use of Tisseel.

Since the introduction of the rhytidectomy (facelift) more than 100 years ago, hematoma formation has persisted as its most common complication. 1 A variety of techniques and products have been used to attempt to address this age-old problem, including compression dressings and surgical drains.

Fibrin tissue sealants have now also been in existence for more than 100 years. 2 Their properties act as a hemostat, sealant, and adhesive. More recently, they have seen a resurgence in use, starting with sutureless microanastamosis in 1972. 3 Their hemostatic and adhesive properties made them a logical choice to address hematoma, and postoperative edema and ecchymosis in facelifts. Bruck performed a pilot study using fibrin tissue adhesion in facelifts in 1982, demonstrating diminished postoperative edema and reduced operative time. 4 Since then, the use of fibrin tissue sealants has increased in facelift. Previous studies, reviews, and anecdotal reports have provided a wide range of results and conclusions on the use of fibrin tissue sealants in facelift. 5 6 7 8 9 10 11 12

Tisseel (Baxter) was approved by the U.S. Food and Drug Administration (FDA) in 1998 for use in hemostasis and wound sealing in surgery. 13 While not designed specifically for facelifts, surgeons learned to modify the mixing process of Tisseel to create a slow-setting sealant and allow for practical use during fixation of the skin flap. 11

Increasing numbers of facial plastic surgeons using Tisseel behooved development of a fibrin tissue sealant designed specifically for facelifts. In 2011, Artiss (Baxter) was FDA approved as the first fibrin tissue sealant for use in facelifts. 14 The low concentration of thrombin (4.5 IU) makes Artiss an adhesive, not a hemostat, and provides the surgeon up to 60 seconds to position the skin flap prior to fixation. 11

In our practice, Tisseel was used in facelifts for more than a decade. We mixed the lyophilized thrombin powder with fibrinogen, aprotinin, and calcium chloride to create a tissue sealant well-suited for facelift flap adhesion. We found the product to be safe and efficacious in minimizing postoperative complications. Incidence of any significant complications including hematoma, seroma, or flap necrosis has been extremely rare. In addition, use of Tisseel simplified postoperative care and improved patient satisfaction. The lack of surgical drains obviated the need for postoperative removal. This has anecdotally improved patient comfort and compliance in the immediate postoperative period. Without a drain to manage, same-day care at home on postoperative day 0 consists only of rest and recovery.

In 2014, our practice switched from Tisseel to Artiss for every facelift performed. The goal of switching products was to reduce the time required for preparation as well as to eliminate potential errors or complications in mixing and preparing the product. After performing 60 facelifts with Artiss, we compared our experience between the two products.

The purpose of this study is to determine the efficacy and safety of Artiss for use in facelifts. In addition, the study will also determine the use of fibrin tissue sealants by facial plastic surgeons.

## Methods/Material

In total, 120 patients in our practice were identified who underwent a facelift from August 2013 to January 2015 by a single surgeon (LMD). The practice made the switch from using Tisseel to Artiss on every facelift in April 2014. The last 60 consecutive patients who underwent facelift with Tisseel before the switch were identified, and the first 60 consecutive patients who underwent facelift with Artiss after the switch were identified. All consecutive patients were included. No other changes in technique, instrumentation, or procedure were made throughout the study period.

To minimize bleeding risk, all patients were required to discontinue any nonsteroidal anti-inflammatory drugs, vitamins, or herbal supplements, which are known anticoagulants, for a minimum of 2 weeks before surgery. A comprehensive list of these medications is provided to every patient during the preoperative visit. In addition, all patients were required to abstain from smoking at least 4 weeks before surgery.

**Fig. 1:**
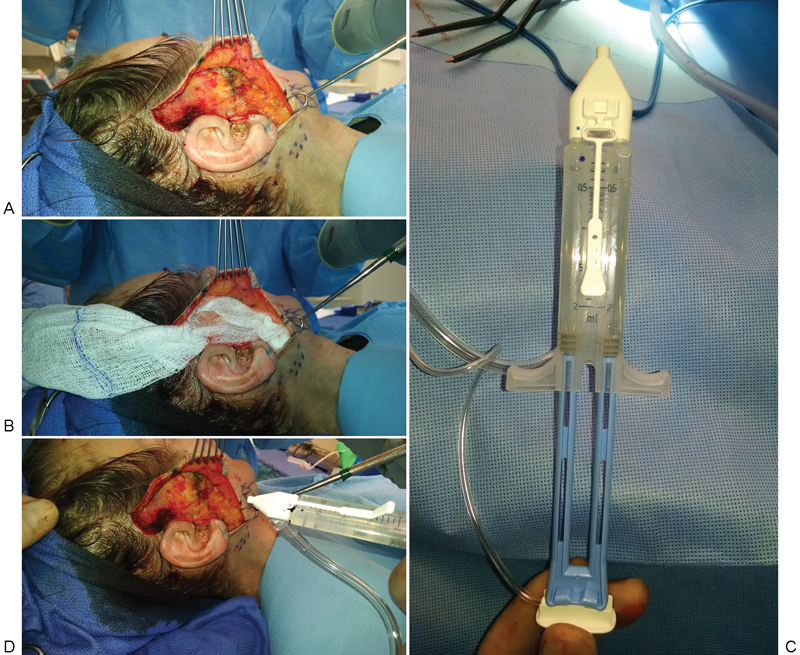
( **A** ) The dissection bed is thoroughly examined, and any bleeding is carefully controlled with bipolar cautery. ( **B** ) The dissection bed is dried with gauze. ( **C** ) The prepackaged Artiss is connected to a nitrogen pressure regulator to create an aerosolized spray. ( **D** ) Approximately 4 to 5 mL of Artiss is sprayed onto the dissection bed. The skin flap is put back down, and light pressure is held for 3 minutes.

As a brief overview of technique, good hemostasis of the subcutaneous dissection bed under the skin flap is achieved with bipolar cautery. The area is then dried with gauze. The fibrin sealant is then sprayed onto the dissection bed using a nitrogen-propelled aerosolizer (Baxter). A thin layer, approximately 4 to 5 mL, is applied to the dissection bed. The skin flap is then placed back down onto the dissection bed, and light pressure is applied for 3 minutes to ensure proper setting of the fibrin sealant ( Fig. 1 ). The incisions are then closed with minimal manipulation of the skin flap. A light cotton dressing is placed over the entire dissection area and left on overnight. The dressing is removed on postoperative day 1.

All perioperative or postoperative complications were identified in the charts and recorded. Complications recorded included hematoma, fluid collection requiring needle aspiration, seroma, flap necrosis, infection, and nerve injury. Proportions were compared using *Fisher's exact two-tailed test* . In addition, the amount of fluid aspirated was recorded, and the total amount aspirated was compared using *Wilcoxon rank-sum test* .

In review of the perioperative records, the overall supply cost and time needed to prepare and deliver Tisseel and Artiss were recorded. Any cases that required more than one package of Tisseel or Artiss due to product inconsistency or device malfunction was recorded, and the additional cost was calculated.

In addition, a six-question survey was created and sent to all members of the American Academy of Facial Plastic and Reconstructive Surgery (AAFPRS), an association of more than 2,700 facial plastic and reconstructive surgeons. The survey asked about the surgeon's level of experience and whether or not fibrin tissue sealant was used during facelift. Others questions asked the surgeon's opinion on the advantages and disadvantages of using fibrin tissue sealant in surgery. The survey was announced through e-mail communication and administered through the online service SurveyMonkey.

Results of the survey were recorded and analyzed for trends or patterns in the data. A free-response question in the survey was also reviewed to obtain surgeons' opinions on the use of fibrin tissue sealant. Institutional review board approval was obtained for this study, including chart review and administration of the survey.

## Results

A total of 120 patients were included in the study. The female-to-male ratio in the Tisseel and Artiss groups was 54 to 6 and 55 to 5, respectively. The average age in the Tisseel and Artiss groups was 63 and 60 years old, respectively.

In the Tisseel group, two complications of fluid collection requiring needle aspiration were recorded with totals of 2 and 7.5 mL, respectively. No other complications were found. In the Artiss group, 10 complications were recorded, including 9 fluid collections requiring needle aspiration with totals of 0.5, 2, 2, 2, 0.5, 10, 1, 3, and 4 mL, respectively. One hematoma was recorded. The needle aspirations ranged from 0.5 to 10 mL and averaged 2.78 mL per aspiration. The difference between two needle aspirations in the Tisseel group and nine needle aspirations in the Artiss group was statistically significant ( Table 1 ). The hematoma required partial opening of the suture line, suctioning under the skin flap, and placement of a light pressure dressing. After regular follow-up, the patient healed completely without further complications and achieved an excellent cosmetic result. No other complications, including skin necrosis, infection, or nerve injury, were found.

**Table 1:** Complications of fibrin tissue sealants

Complication	Tisseel	Artiss	*p* -Value
Fluid aspiration	2	9	0.05
Fluid per aspiration (mL)	4.75	2.78	0.28
Hematoma	0	1	–
Total complication	2	10	0.02

The perioperative review of Tisseel and Artiss at our institution was shown to have an average supply cost difference of $10 per 10 mL package of product. The average time of preparation for Tisseel was 20 minutes, which included mixing and delivering the product to the field by the nurse and surgical technique. Specific to our facility, 20 minutes of operating room time translated into approximately $200 in facility fees. The average time of preparation for Artiss was 30 seconds. In the Tisseel group, six cases required use of an additional package of product due to mixing inconsistency. No additional packages were needed in the Artiss group.

In total, 179 members of the AAFPRS completed the six-question survey. The survey was administered over a 3-month period from December 2014 through February 2015. A good distribution of experience was seen for both the number of years in practice and the average number of facelifts performed per year ( Table 2 ). Of all respondents, 61 (34%) use tissue sealants for facelifts, whereas 118 (66%) do not. A good distribution was seen based on experience level ( Table 3 ).

**Table 2:** Distribution of survey responses based on experience

Years in practice	0–5 y	6–10 y	11–20 y	21–30 y	30+ y
Total	39	30	46	45	19
Facelifts per year	0–10	10–30	31–50	51–100	100+
Total	53	55	34	22	15

**Table 3:** Fibrin tissue sealant use based on experience

Years in practice	0–5 y	6–10 y	11–20 y	21–30 y	30+ y	Total
Use fibrin sealant	10	8	15	17	11	61
No fibrin sealant	29	22	31	28	8	118
Facelifts per year	0–10	10–30	31–50	51–100	100+	Total
Use fibrin sealant	11	22	12	9	7	61
No fibrin sealant	42	33	22	13	8	118

Of those respondents who did not use fibrin sealant, 44 (25%) used surgical drains (40%) and 67 (60%) did not use any product during closure. Overall, the majority (39%) of respondents report using no product for facelifts.

The perceived advantages of fibrin sealant most cited were no need for surgical drains (59%), ease of use (47%), reduced hematoma, edema, or fluid collection (47%), and simplified postoperative care (41%). The perceived disadvantages of fibrin sealant most cited were increased cost (90%), potential infection or allergic response (33%), need for OR staff education (33%), and lack of availability (23%).

## Discussion

In this study, we evaluated the safety and efficacy of Artiss, the only tissue sealant FDA approved specifically for facelift. To our knowledge, this is the only large, independent clinical study of its kind. The recent switch from Tisseel to Artiss in our practice was a unique opportunity to directly compare the two products by use of a single surgeon with the same surgical technique. Though both products are similar in composition, Tisseel requires premixing and preparation before use. This brings in added time and cost to prepare the product and theoretical risks of mixing or dosing errors. In contrast, Artiss comes in a ready-to-use package and requires only thawing before use.

In our experience, Tisseel is safe and efficacious for facelift. In this study, we have demonstrated that Artiss also has a good safety and efficacy profile. In prior clinical trials, Hester et al demonstrated that Artiss is safe and efficacious and reduces postoperative drainage volume compared with no sealant use. 15

When analyzing the differences in complication rate between the two products, we saw a statistically significant increase in fluid collection requiring aspiration with Artiss. We also considered this clinically significant, as we had seven more patients during the study period with postoperative fluid collections that required drainage with Artiss. This may indicate that the ability of Artiss to seal the skin flap to underlying tissue may be somewhat decreased compared with Tisseel. Anecdotally, the primary surgeon noticed less “stick” of the flap after application of Artiss. Keeping this in mind, the relative adhesive ability of the two products may or may not be a factor in the increased postoperative fluid collection. Overall, complications were minimal, and no other significant differences were noted between the two products.

Perioperative evaluation of supply cost showed a minimal difference between Artiss and Tisseel. The main difference between the two products is demonstrated in the amount of time needed to prepare the two products. The OR nurse and surgical technique required 20 minutes, on average, to prepare Tisseel. Artiss requires only opening of the package. The extra staff time needed as well as the potential for mixing errors highlights the main advantage of Artiss over Tisseel. In our experience, the gained time and ease of using Artiss have improved the efficiency of facelifts since making the switch.

While the development and use of fibrin tissue sealants for facelift has progressed throughout recent years, the product has not been adopted consistently throughout the facial plastic surgery community. Indeed, in terms of prevention of hematoma, the varied results for any adjunctive technique has failed to identify any single best practice for facelift. 1 In addition, newer techniques have also been described as options, including external quilting sutures. 16 We administered a survey to all AAFPRS members to better understand its use or lack thereof.

Experience did not appear to be a factor in whether or not a surgeon used a fibrin sealant. Of the 34% of those surgeons who used fibrin sealant, a good distribution was seen across both years of experience and average number of facelifts performed per year. Overall, the majority of surgeons used no product or surgical drains when performing a facelift. Surgical drains have been demonstrated to reduce the rate of seroma formation and ecchymosis. 17 18

In terms of the perceived advantages of fibrin sealants, the obviated need for a surgical drain was the most cited, followed by ease of use and reduced hematoma, fluid collection, or edema. Kamer and Nguyen showed decreased postoperative edema, induration, and ecchymosis. 7 Several studies have shown a significant decrease in postoperative fluid collection, whereas other studies showing the difference in rate of hematoma are equivocal. 5 9 15 19

The overwhelmingly cited disadvantage of fibrin sealants is increased cost (90%). The cost of fibrin sealants is not insignificant and can cut into the bottom line. The current list price for 10 mL of Artiss is $880.68. Fezza et al stated that some reduction in operative time can partially offset the added expense of the product. 20 In general, cost and accessibility of fibrin sealants is a consideration in its use.

Reviewing the free responses to the survey, one surgeon stated he would never do a facelift without fibrin sealant. Another surgeon stated that the lack of difference in overall outcome does not justify increased cost of the product. Other free responses covered a spectrum of opinions regarding fibrin sealants; however, no trends were found in the responses to make any conclusions.

We argue that the reason is not in the overall outcome but rather in the immediate postoperative recovery experience of the patients. In our practice, we promote a reduced recovery time facelift surgery, including ease of wound care and reduced bruising and swelling. We find this to be appealing to and sought out by the vast majority of patients. Without the need of a heavy pressure dressing or removal of surgical drains, we have simplified care and improved comfort for the patient. Simplified postoperative care was also a commonly cited advantage of tissue sealants in our survey.

Finally, other perceived disadvantages of fibrin sealants are lack of availability (23%) and need for OR staff education about the product (33%). Though fibrin sealants have been used in facelifts for many years, the lack of ubiquity has not yet made them readily accessible to all surgeons, depending on the location and type of surgical center. Further studies and education about these products are needed to expand their use in facial cosmetic surgery.

As previously discussed, Artiss is a human blood-derived product. Such products carry a theoretical risk of transmission of hepatitis B and C, parvovirus, human immunodeficiency virus, and human T-cell leukemia virus. There is also a risk of allergic reaction. These potential risks were cited as a disadvantage by 33% of survey respondents.

Baxter sources plasma products solely from the United States, using its own plasmapheresis facilities. Patients from low-risk populations are screened using questionnaires. The blood products are held for 60 days and then tested for HIV, hepatitis A, B, and C, and parvovirus B19. The products are then treated to inactivate any possible viruses. 21

To our knowledge, no cited reports exist of transmission of blood-borne pathogens with fibrin sealant. In addition, previous studies have demonstrated blood transfusions to be very safe since the advance in screening and processing of blood products since the late 1980s. 22

Weaknesses of this study include a lack of a control group without fibrin tissue sealant use, though we wanted to directly compare two products we use consistently in our facelifts. In addition, this was a retrospective chart review, and the surgeon was not blinded to the treatment. Finally, our study was limited to a single practice and single surgeon, though this helped maintain consistency between treatment groups.

In this study, we have demonstrated that Artiss is efficacious. Its use obviates the need for surgical drains, and complications are minimal and similar in rate to the use of Tisseel. There was, however, a significant increase in fluid aspirations, which could be related to the adhesive strength to the product. Artiss is also shown to be safe. No adverse events linked directly to use of the product were found. The use of Artiss in facelift surgery can be an effective tool in simplifying care and improving the immediate postoperative healing experience of the patient.
